# Tectorigenin protects against experimental fulminant hepatic failure by regulating the TLR4/mitogen‐activated protein kinase and TLR4/nuclear factor‐κB pathways and autophagy

**DOI:** 10.1002/ptr.6299

**Published:** 2019-01-30

**Authors:** Lingjian Zhang, Yalei Zhao, Linxiao Fan, Kai Xu, Feiyang Ji, Zhongyang Xie, Xiaoxi Ouyang, Daxian Wu, Lanjuan Li

**Affiliations:** ^1^ State Key Laboratory for Diagnosis and Treatment of Infectious Diseases, Collaborative Innovation Center for Diagnosis and Treatment of Infectious Diseases, The First Affiliated Hospital Zhejiang University School of Medicine Hangzhou China; ^2^ Department of Orthopaedic Surgery, Second Affiliated Hospital Zhejiang University School of Medicine Hangzhou China

**Keywords:** autophagy, fulminant hepatic failure, inflammation, tectorigenin

## Abstract

Tectorigenin has received attention due to its antiproliferation, anti‐inflammatory, and antioxidant activities. In this study, we investigated the effects of tectorigenin on lipopolysaccharide (LPS)/D‐galactosamine(D‐GalN)‐induced fulminant hepatic failure (FHF) in mice and LPS‐stimulated macrophages (RAW 264.7 cells). Pretreatment with tectorigenin significantly reduced the serum levels of alanine aminotransferase (ALT) and aspartate aminotransferase (AST), histological injury, apoptosis, and the mortality of FHF mice, by suppressing the production of inflammatory cytokines such as TNF‐α and IL‐6. Tectorigenin also suppressed the activation of the inflammatory response in LPS‐stimulated RAW 264.7 cells. Tectorigenin‐induced protection is mediated through its mitigation of TLR4 expression, inhibition of mitogen‐activated protein kinase (MAPK) and nuclear factor‐κB (NF‐κB) pathway activation, and promotion of autophagy in FHF mice and LPS‐stimulated RAW 264.7 cells. Therefore, tectorigenin has therapeutic potential for FHF in mice via the regulation of TLR4/MAPK and TLR4/NF‐κB pathways and autophagy.

## 1 INTRODUCTION

Fulminant hepatic failure (FHF) is a fatal clinical syndrome induced by drug‐induced liver injury, ischemic and pregnancy‐associated liver injury, acute infection with hepatitis A or B virus, autoimmune hepatitis, Budd–Chiari syndrome, and Wilson disease, resulting in severe liver injury (Bernal, Lee, Wendon, Larsen, & Williams, 2015; Ostapowicz et al., 2002). FHF can lead to hepatic encephalopathy, systemic inflammatory response syndrome, and ultimately multiorgan failure (Bernal et al., 2015). Hepatocyte death and activation of hepatic inflammation are two important features of FHF (Antoniades, Berry, Wendon, & Vergani, 2008; Bernal et al., 2015; Malhi, Guicciardi, & Gores, 2010).

Hepatic inflammation is involved in the pathogenesis of FHF (Antoniades et al., 2008). The liver is an immune organ enriched in immune cells such as Kupffer cells (hepatic macrophages; Crispe, 2009; Malhi et al., 2010). Upon receiving inflammatory signals, the Kupffer cells are activated, recruiting monocytes and macrophages (Wu, Han, Chen, Yan, & Ning, 2010). These immune cells contribute to inflammation and aggravate liver injury (Malhi et al., 2010). Lipopolysaccharide (LPS)‐ and D‐galactosamine (D‐GalN)‐induced FHF is a frequently used animal model that closely mimics endotoxemia‐induced clinical FHF (Ewaschuk et al., 2007). Toll‐like receptor 4 (TLR4) is an important LPS receptor, and TLR4‐mediated mitogen‐activated protein kinase (MAPK) and nuclear factor‐κB (NF‐κB) signaling pathways play important roles in inflammation and FHF (Crispe, 2009; Malhi et al., 2010). Autophagy is a homeostatic degradative process that removes damaged organelles and turns over cytoplasmic factors in eukaryotic cells (Klionsky & Emr, 2000). It was reported that autophagy may protect against FHF by regulating immune responses (Czaja et al., 2013).

**Figure 1:**
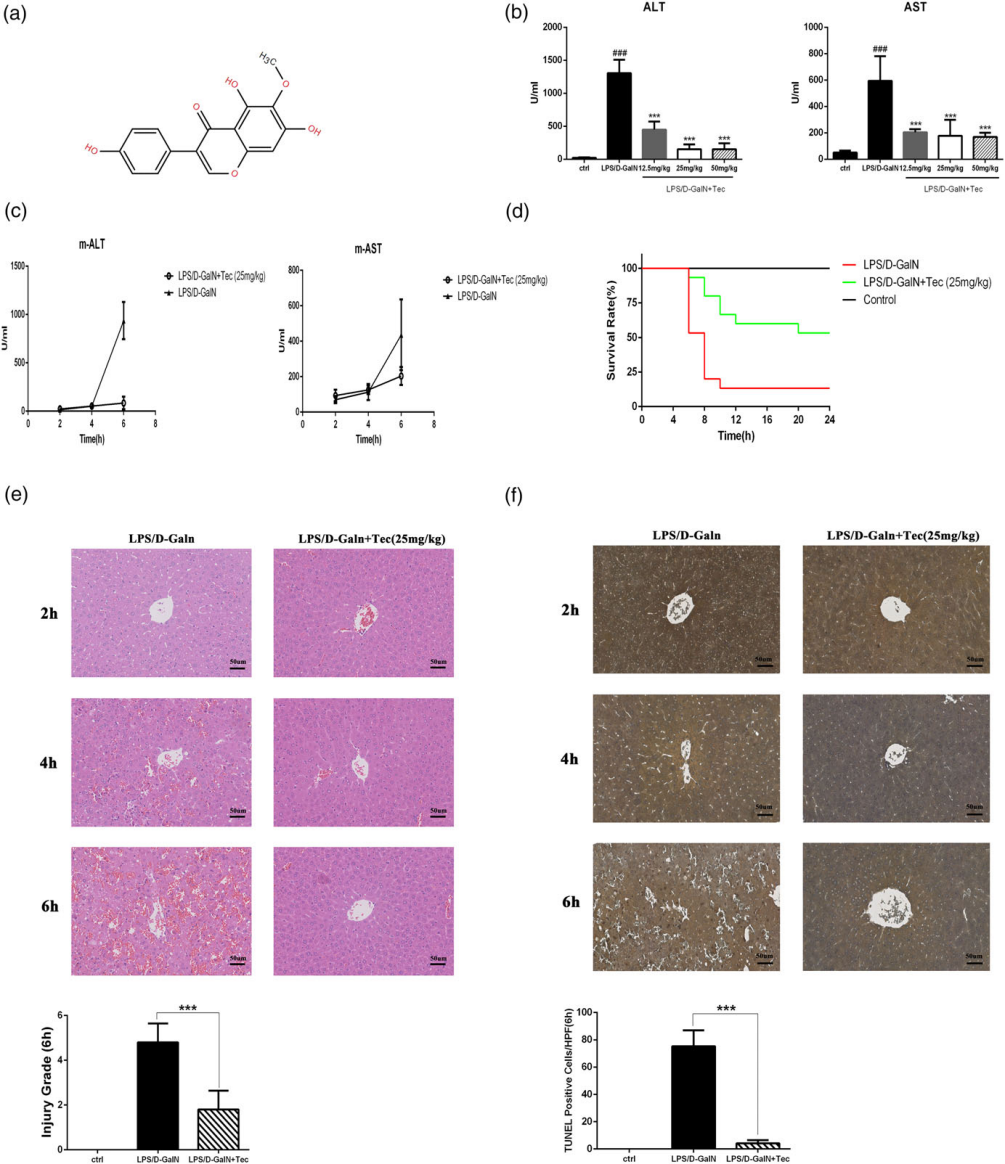
Tectorigenin (Tec) prevents LPS/D‐GalN‐induced liver injury (a) chemical structure of Tec. (b) Serum AST and ALT levels in fulminant hepatic failure mice pretreatment with different concentrations of Tec (12.5, 25, and 50 mg/kg) at 6 hr (*n* = 5). (c) Serum AST and ALT levels in mice at 2, 4, and 6 hr (*n* = 5). (d) Survival rate of LPS/D‐GalN‐induced mice after pretreatment with phosphate buffered saline or Tec for 24 hr (*n* = 15). (e) Hematoxylin–eosin‐stained liver sections (magnification, 200×). Histological grades of liver injury at 6 hr (*n* = 5). (f) Images of TUNEL‐stained mouse livers with the treatment as shown (magnification, 200×). TUNEL‐positive cells per high‐power field counted at 6 hr (*n* = 5). (*p < 0.05, **p < 0.01, and ***p < 0.001 compared with the LPS/D‐GalN group at the same time point. #*p* < 0.05, ##*p* < 0.01, and ###*p* < 0.001 compared with the control group). LPS/D‐GalN: lipopolysaccharide/D‐galactosamine‐GalN [Colour figure can be viewed at wileyonlinelibrary.com]

Tectorigenin (Tec; Figure 1a), a component of Belamcanda Adans, has antiproliferation, anti‐inflammatory, and antioxidant activities (Wang et al., 2017). Presently, the main studies on Tec have focused on its antitumor effects via the NF‐κB and MAPK pathways (Yang et al., 2012; Zeng et al., 2018). Tec was also reported to demonstrate a protective effect on acute lung injury (Ma, Liu, Qu, & Ma, 2014) and hepatoprotective effects in models of chemical and oxidative damage (H. U. Lee, Bae, & Kim, 2005; H. W. Lee, Choo, Bae, & Kim, 2003). However, little information is available to reveal the therapeutic potential of Tec against a fulminant and deadly hepatic failure model. To the best of our knowledge, the effect of Tec on autophagy also remains unknown. Therefore, we investigated the therapeutic potential of Tec against FHF.

## 2 MATERIALS AND METHODS

### 2.1 Materials

Tec (C_16_H_12_O_6_, purity >98%, MW = 300.26) was purchased from Feiyu Biotechnology Corporation (Nantong, China). The In Situ Cell Death Detection Kit, LPS, and D‐GalN were obtained from Sigma‐Aldrich (St Louis, MO, USA). The NF‐κB Pathway Sampler Kit, MAPK Family Antibody Sampler Kit, and goat anti‐mouse IgG‐horseradish peroxidase (HRP) and goat anti‐rabbit HRP were purchased from Cell Signaling Technology (Beverly, MA, USA). Antibodies against GAPDH, LC3 I, LC3 II, Histone H3, and P62 were from Abcam (Cambridge, UK). The DAB substrate kit was from Abcam. The ALT and AST kits were purchased from Jiancheng Bioengineering Institute (Nanjing, China). The bicinchoninic acid protein assay kit, RIPA, 5× loading buffer, QuickBlock™ Western Blocking Buffer, and phosphatase and protease inhibitor cocktails were purchased from Shanghai Beyotime Biotechnology Corporation (Shanghai, China). The IL‐6 and TNF‐α enzyme‐linked immunosorbent assay kits were from eBioscience (San Diego, CA, USA). The CCK8 assay kit was obtained from Dojindo (Kumamoto, Japan).

### 2.2 Animals

Male C57BL/6J mice (weighing 20–25 g) were obtained from Beijing Vital River Laboratory Animal Technology Corporation. All animal experiments were approved by the Ethics Committee of the First Affiliated Hospital, Zhejiang University School of Medicine, and were performed in accordance with the Interdisciplinary Principles and Guidelines for the Use of Animals in Research, Testing, and Education of the Ad Hoc Animal Research Committee, New York Academy of Sciences. Liver injury was induced by the intraperitoneal injection of 50 μg/kg LPS and 800 mg/kg D‐GalN (Sigma) in phosphate‐buffered saline (PBS) as reported previously (Lin et al., 2014). Tec was dissolved in PBS containing 5% dimethyl sulfoxide. For pretreatment, an equal volume of PBS with 5% dimethyl sulfoxide or different concentrations of Tec (12.5, 25, and 50 mg/kg) was injected intraperitoneally 30 min before LPS/D‐GalN administration. To observe the survival of different groups, 45 mice (15 mice per group) were used. After LPS/D‐GalN injection, mortality was recorded every 2 hr, and the survival rates were continuously monitored for 24 hr. After 24 hr, 2 mice were left in LPS/D‐GalN group, 8 mice in LPS/D‐GalN+Tec (25 mg) group, and 15 mice in control group. All mice were anesthetized with isoflurane inhalation. For some experiments (isolation of liver tissue and blood), mice (five mice per group) were sacrificed at 2,4, and 6 hr after the GalN/LPS injection. Blood was collected from the retroorbital venous plexus and was centrifuged at 4°C for 10 min at 3,500 rpm. The livers were washed twice with distilled PBS, and a portion was used for histopathology. The remaining tissue was cryopreserved to prepare the liver homogenates.

### 2.3 Determination of liver enzyme activities

Serum was collected and stored at −80°C until use. ALT and AST activities were determined using commercial kits (Jiancheng) following the manufacturer's instructions.

### 2.4 Histology

The left lobes of the liver tissue samples were fixed with 4% paraformaldehyde for 24 hr, embedded in paraffin, and cut into 5‐μm‐thick sections. The sections were stained with hematoxylin–eosin, and representative images of nonconsecutive free‐selection 200× histological fields were captured using a digital slice scanning equipment (NanoZoomer 2.0RS). Hematoxylin–eosin stained livers were evaluated by a single pathologist in a blinded fashion on the reported semiquantitative scale (Perego et al., 2017).

The total score for acute hepatitis is given by the sum of the inflammation score (portal + lobular scores) and necrosis score, which are scored as follows.

Inflammation score: 0, no inflammation; 1, mild lobular inflammation (<10% of liver parenchyma)/mild portal inflammation (<1/3 of portal tracts); 2, moderate lobular inflammation (10–50% of liver parenchyma)/moderate portal inflammation (approximately 50% of portal tracks); and 3, severe lobular inflammation (>50% of liver parenchyma)/severe portal inflammation (>2/3 of portal tracts). Necrosis score: 0, no necrosis; 1, <10% necrosis of liver parenchyma; 2, 10–25% necrosis of liver parenchyma; and 3, >25% necrosis of liver parenchyma.

Apoptotic cells were detected by TUNEL staining using an In Situ Cell Death Detection Kit (Sigma) and a DAB Substrate Kit (Abcam). The number of TUNEL‐positive cells were counted and recorded in five randomly selected high‐power fields (magnification, 200×) per liver section.

### 2.5 Cell culture

RAW 264.7 murine macrophages and immortalized normal human liver LO2 cells were cryopreserved at the State Key Laboratory for Diagnosis and Treatment of Infectious Diseases, Zhejiang University. RAW 264.7 cells were cultured in Dulbecco's modified Eagle's medium (DMEM) (Gibco) supplemented with 100 IU/ml of penicillin, 100 μg/ml streptomycin, and 10% heat‐inactivated fetal bovine serum. LO2 cells were cultured in DMEM supplemented with 100 IU/ml penicillin, 100 μg/ml streptomycin, and 10% fetal bovine serum. The cells were incubated at 37°C in a fully humidified incubator with a 5% CO_2_ atmosphere and were subcultured twice weekly. RAW 264.7 cells were seeded at 5 × 10^5^/well in 12‐well tissue culture plates, incubated for 12 hr at 37°C in a fully humidified incubator with a 5% CO_2_ atmosphere, and treated with LPS (100 ng/ml) and Tec simultaneously. The control group was treated with DMEM. RAW 264.7 cells were harvested at 6 hr for quantitative real‐time reverse transcription‐polymerase chain reaction and Western blotting.

### 2.6 Cell proliferation assay

RAW 264.7 cells and LO2 cells were seeded at 1 × 10^4^/well in 96‐well tissue culture plates and treated with Tec for 24 hr. Cell viability was measured over 2 hr by CCK8 quantitative colorimetric assay (Dojindo) according to the manufacturer's instructions.

### 2.7 Quantitative real‐time reverse transcription‐polymerase chain reaction

Total RNA was isolated from cultured cells and liver tissue using TRIzol (Invitrogen) according to the manufacturer's instructions and was reverse transcribed into cDNA using the PrimeScript™ RT Master Mix (TaKaRa), and primers (Table S1) were purchased from Generay Biotechnology Corporation (Shanghai, China). PCR was conducted using a 7500 Real‐Time PCR system (Applied Biosystems). Data analyses were performed using the 2^‐ΔΔCT^ method for relative quantification using β‐actin as a reference gene for normalization.

### 2.8 Cytokine assays

The cytokine levels of IL‐6 and TNF‐α were measured using enzyme‐linked immunosorbent assay kits (eBioscience) according to the manufacturer's instructions.

### 2.9 Western blotting

For total protein isolation, portions of the liver were sonicated in RIPA lysis solution containing protease and phosphatase inhibitors (Beyotime). The protein in cytosol and nucleus was extracted using the Minute™ Cytoplasmic and Nuclear Fractionation kit (Inventbiotech, Beijing, China) according to the manufacturer's protocol. The cells were washed three times with PBS, centrifuged, and lysed with RIPA containing phosphatase and protease inhibitor cocktails. The protein concentrations of the lysates were measured using the bicinchoninic acid assay. The protein samples (50 μg) were mixed with 5× loading buffer (Beyotime), boiled for 10 min, separated on a 12% or 15% (*w*/*v*) sodium dodecyl sulfate‐polyacrylamide gel electrophoresis gel (Beyotime) and transferred to a polyvinylidene difluoride membrane. The membrane was blocked with QuickBlock™ Western Blocking Buffer (Beyotime) for 0.5 hr and was incubated with the appropriate primary antibodies overnight at 4°C. The membranes were next incubated for 1.5 hr with the appropriate HRP‐conjugated secondary antibodies. Target proteins were visualized using a chemiluminescence imaging system (Clinx Science Instruments). The protein levels were normalized to those of GAPDH or Histone H3.

### 2.10 Transmission electron microscope evaluation

Liver tissues (<5 mm) were soaked in 2.5% glutaraldehyde in 0.1 M PBS at 4°C overnight and post‐fixed in 1% OsO4 in 0.1 M PBS at room temperature for 1 hr. The sample was first dehydrated in graded alcohols, transferred to absolute acetone for 20 min, infiltrated in mixture of absolute acetone and Spurr resin, and then embedded in Spurr resin for sectioning. Ultrathin (70–90 nm) sections were collected on nickel grids. Images were acquired with a digital electron microscope (H‐7650, Hitachi Ltd, Tokyo, Japan).

### 2.11 Statistical analyses

The data were expressed as the means ± standard deviation of at least three independent experiments. The statistical significance of differences between groups was assessed by one‐way analysis of variance using SPSS 18.0 software (SPSS Inc., Chicago, IL, USA). The survival rates were compared by Kaplan–Meier analyses and the log‐rank test. Statistical significance was defined as *p* < 0.05.

## 3 RESULTS

### 3.1 Tectorigenin ameliorates lipopolysaccharide/D‐galactosamine‐GalN‐induced fulminant hepatic failure

We first investigated whether pretreatment with different concentrations of Tec (12.5, 25, and 50 mg/kg) has protective effects on LPS/D‐GalN‐induced FHF. We injected C57BL/6J mice with different concentrations of Tec or PBS 30 min prior to LPS/D‐GalN administration. Regarding liver injury, the serum levels of ALT and AST were significantly increased in the LPS/D‐GalN group at 6 hr (Figure 1b,c). Tec dramatically reduced the serum levels of ALT and AST at 6 hr (Figure 1b,c); 25 mg/kg of Tec treated group has lower level of ALT than 12.5 mg/kg Tec treated group, but there were no significant differences between 25 and 50 mg/kg treatments. (Figure 1b). Thus, we chose the concentration 25 mg/kg in the following in vivo experiment. We further investigated the effect of Tec on the survival rate of FHF mice. Mice in the LPS/D‐GalN‐treated group started to die at 6 hr, and the mortality was 87% at 10 hr. Tec pretreatment significantly decreased the mortality (Figure 1d). Mice in the LPS/D‐GalN group showed disrupted liver architecture, vacuolization with disappearance of nuclei, necrosis, and severe tissue hemorrhage; these changes were reversed by Tec (Figure 1e). The number of terminal deoxynucleotide transferase deoxyuridine triphosphate nick end‐labeling (TUNEL)‐positive (apoptotic) hepatocytes at 6 hr was increased in the LPS/D‐GalN group and was significantly decreased by Tec (Figure 1f). Therefore, Tec pretreatment markedly inhibited liver injury and improved survival of FHF mice.

### 3.2 Tectorigenin reduces the cytokine levels of lipopolysaccharide/D‐galactosamine‐GalN‐induced mice

**Figure 2:**
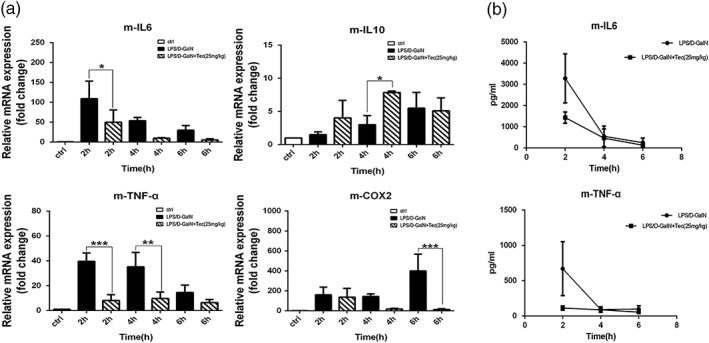
Effects of tectorigenin on the cytokine levels of mice (a) IL‐6, IL‐10, TNF‐α, and COX2 mRNA expression determined by quantitative real‐time reverse transcription‐polymerase chain reaction (*n* = 3). (b) Serum levels of TNF‐α and IL‐6 (*n* = 5). (*p < 0.05, **p < 0.01, and ***p < 0.001 compared with the LPS/D‐GalN group at the same time point). LPS/D‐GalN: lipopolysaccharide/D‐galactosamine‐GalN

We assessed the mRNA levels of interleukin 6 (IL‐6), tumor necrosis factor (TNF‐α), interleukin 10 (IL‐10), and cyclooxygenase 2 (COX2) in mice by quantitative real‐time reverse transcription‐polymerase chain reaction. The IL‐6, TNF‐α, and COX2 mRNA levels were significantly increased by LPS/D‐GalN administration (Figure 2a). Tec pretreatment dramatically reduced the IL‐6, TNF‐α, and COX2 mRNA levels (Figure 2a). The mRNA of IL‐10 was significantly increased at 4 hr (Figure 2a). Additionally, the circulating levels of proinflammatory cytokines were significantly increased by LPS/D‐GalN (Figure 2b). Tec application significantly reduced the serum levels of TNF‐α and IL‐6 at 2 hr (Figure 2b). Nevertheless, the protective effects of Tec against FHF may due to the decreased inflammatory cytokine levels.

### 3.3 Tectorigenin prevents liver injury via the TLR4/mitogen‐activated protein kinase and TLR4/nuclear factor‐κB pathways and autophagy

**Figure 3:**
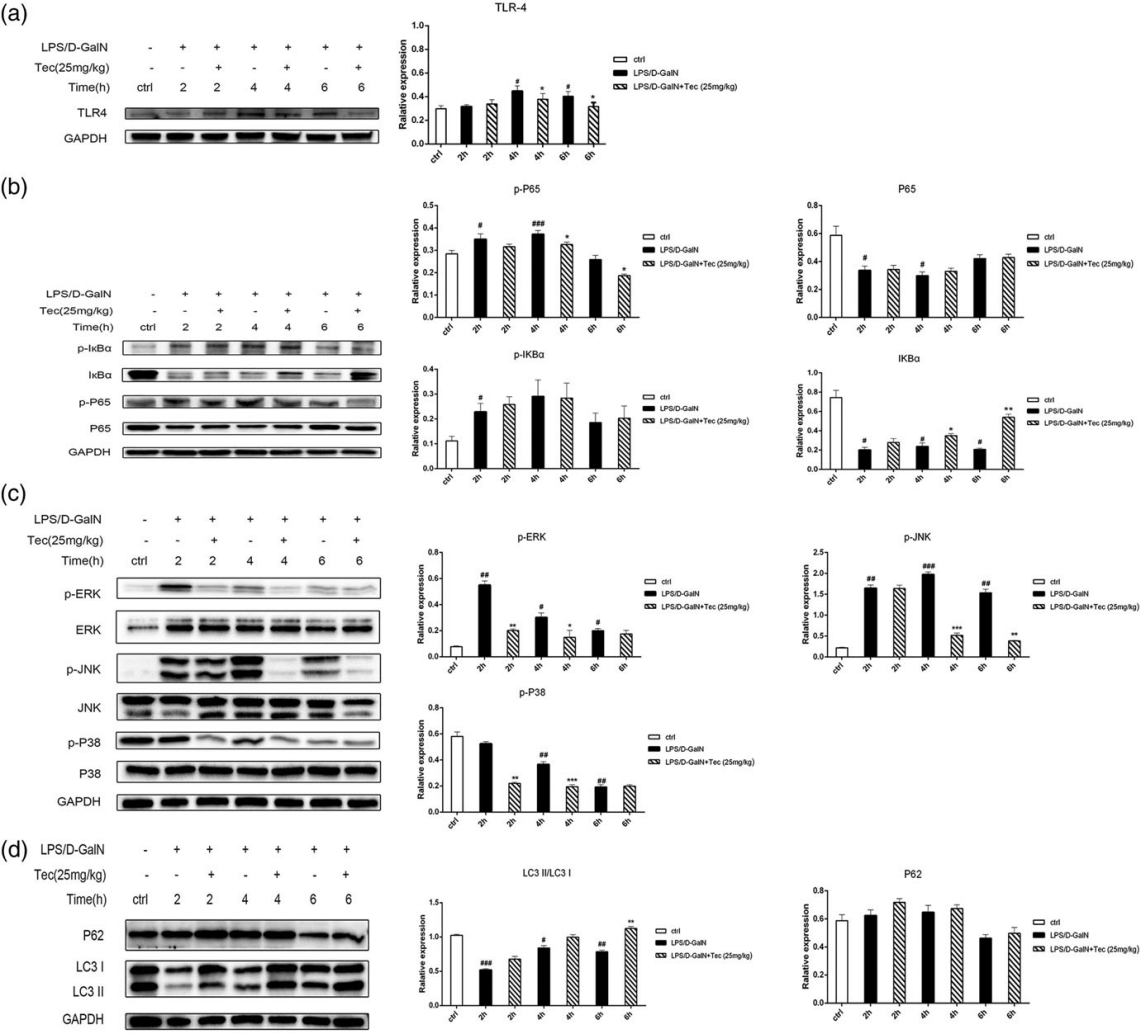
Tectorigenin blocks TLR4/mitogen‐activated protein kinase and TLR4/nuclear factor‐κB pathway activation and promotes autophagy in mice. Levels of (a) TLR4; (b) phosphorylated and total p65 and IκBα; (c) phosphorylated and total ERK, p38, and JNK; and (d) LC3 I, LC3 II, and p62 in liver tissues. (*p < 0.05, **p < 0.01, and ***p < 0.001 compared with the LPS/D‐GalN group at the same time point. #*p* < 0.05, ##*p* < 0.01, and ###*p* < 0.001 compared with the control group; *n* = 3). LPS/D‐GalN: lipopolysaccharide/D‐galactosamine‐GalN; ERK: extracellular signal‐regulated kinase; JNK: JNK: c‐Jun N‐terminal kinase

The activity of TLR4/MAPK and TLR4/NF‐κB signaling pathways is correlated with FHF and inflammation (Crispe, 2009; Malhi et al., 2010). We first detected the expression of TLR4 in the liver tissues of FHF mice; the TLR4 levels were significantly increased in LPS/D‐GalN‐induced FHF mice at 4 and 6 hr (Figure 3a); and Tec significantly decreased the expression of TLR4 in vivo (Figure 3a). We also probed the effect of Tec on the activation of the NF‐κB pathway in vivo. NF‐κB p65 phosphorylation was elevated in liver of LPS/D‐GalN‐induced FHF mice (Figure 3b), and IκBα was significantly downregulated (Figure 3b); thus, the NF‐κB pathway was activated after LPS/D‐GalN administration. However, Tec pretreatment could suppress the activation of the abovementioned pathway. In the Tec pretreatment group, compared with the LPS/D‐GalN group, the phosphorylation of NF‐κB p65 was significantly decreased (Figure 3b), whereas IκBα levels were increased (Figure 3b). Therefore, the protective effects of Tec on FHF mice may be partially mediated through the inhibition of the NF‐κB signaling pathway.

Next, to further illustrate whether the MAPK pathway was involved in the therapeutic effects of Tec, we detected the activation of MAPK pathway in our present study. In the LPS/D‐GalN group, the phosphorylation of extracellular signal‐regulated kinases 1 and 2 (ERK1/2) and c‐Jun N‐terminal kinase (JNK) were significantly increased (Figure 3c). As expected, the activation of the MAPK pathway was alleviated by Tec pretreatment. The phosphorylation of p38, ERK1/2, and JNK were reduced by Tec pretreatment (Figure 3c). Thus, to some extent, FHF mice pretreated with Tec in this study may benefit from inhibition of the MAPK pathway.

Interestingly, we also found that the therapeutic effects of Tec were related to autophagy. Autophagy was suppressed by LPS/D‐GalN in vivo (Figure 3d), a finding that was consistent with the previous published studies. However, Tec pretreatment promoted autophagy as the lipidation of LC3I to LC3 II was accelerated, but the expression of p62 was not significantly influenced (Figure 3d). To further confirm whether Tec could inhibit the FHF by promoting autophagy, we observed autophagosomes by electron microscopy. We found that the number of autophagosomes was significantly increased in the Tec treatment group at 6 hr (Figure S1). Therefore, Tec promotes autophagy in LPS/D‐GalN‐induced FHF.

### 3.4 Effects of tectorigenin on RAW 264.7 and LO2 cell viability and the cytokine levels of lipopolysaccharide‐stimulated RAW 264.7 cells

**Figure 4:**
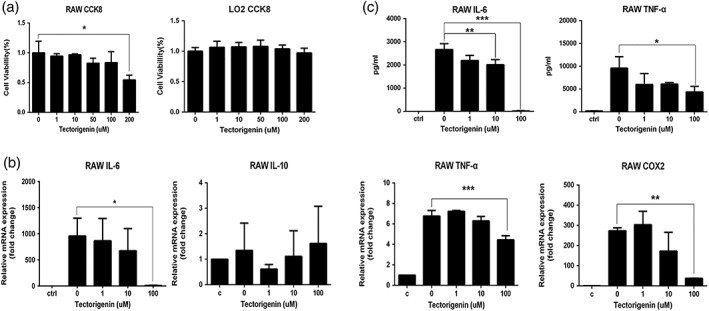
Tectorigenin (Tec) inhibits the activation of the inflammatory response in lipopolysaccharide (LPS)‐stimulated RAW 264.7 cells. (a) RAW 264.7 cells and LO2 cells were pretreated with Tec for 24 hr, and their viability was evaluated by the CCK8 assay (*n* = 3). (b) IL‐6, TNF‐α, IL‐10, and COX2 mRNA levels were determined by quantitative real‐time reverse transcription‐polymerase chain reaction in RAW 264.7 cells treated with Tec and LPS (100 ng/ml) for 6 hr (*n* = 3). (c) IL‐6 and TNF‐α levels in medium measured using enzyme‐linked immunosorbent assay kits (*n* = 3). (*p < 0.05, **p < 0.01, and ***p < 0.001 compared with the LPS group. #*p* < 0.05, ##*p* < 0.01, and ###*p* < 0.001 compared with the control group; *n* = 3)

Next, we investigated the anti‐inflammatory effect and cytotoxic in vitro. The cytotoxic effects of Tec on RAW 264.7 cells were examined using the Cell Counting Kit‐8 (CCK8) assay. Treatment for 24 hr with 1–100 μM of Tec did not affect the cell viability of RAW 264.7 cells (Figure 4a). Thus, 1–100 μM of Tec was used in all subsequent in vitro experiments. We also tested the cytotoxicity of Tec on immortalized normal human liver LO2 cells. Tec also did not show significant hepatotoxicity at the concentration of 1–200 μM of Tec in 24 hr (Figure 4a).

Treatment of RAW 264.7 cells with LPS resulted in the increased mRNA expression of IL‐6, TNF‐α, and COX2, which were significantly reduced by Tec (Figure 4b). Tec suppressed the levels of proinflammatory cytokines IL‐6 and TNF‐α in the culture supernatant in a concentration‐dependent manner (Figure 4c).

### 3.5 Effects of tectorigenin on the TLR4/mitogen‐activated protein kinase and TLR4/nuclear factor‐κB pathways and on autophagy in lipopolysaccharide‐stimulated RAW 264.7 cells

**Figure 5:**
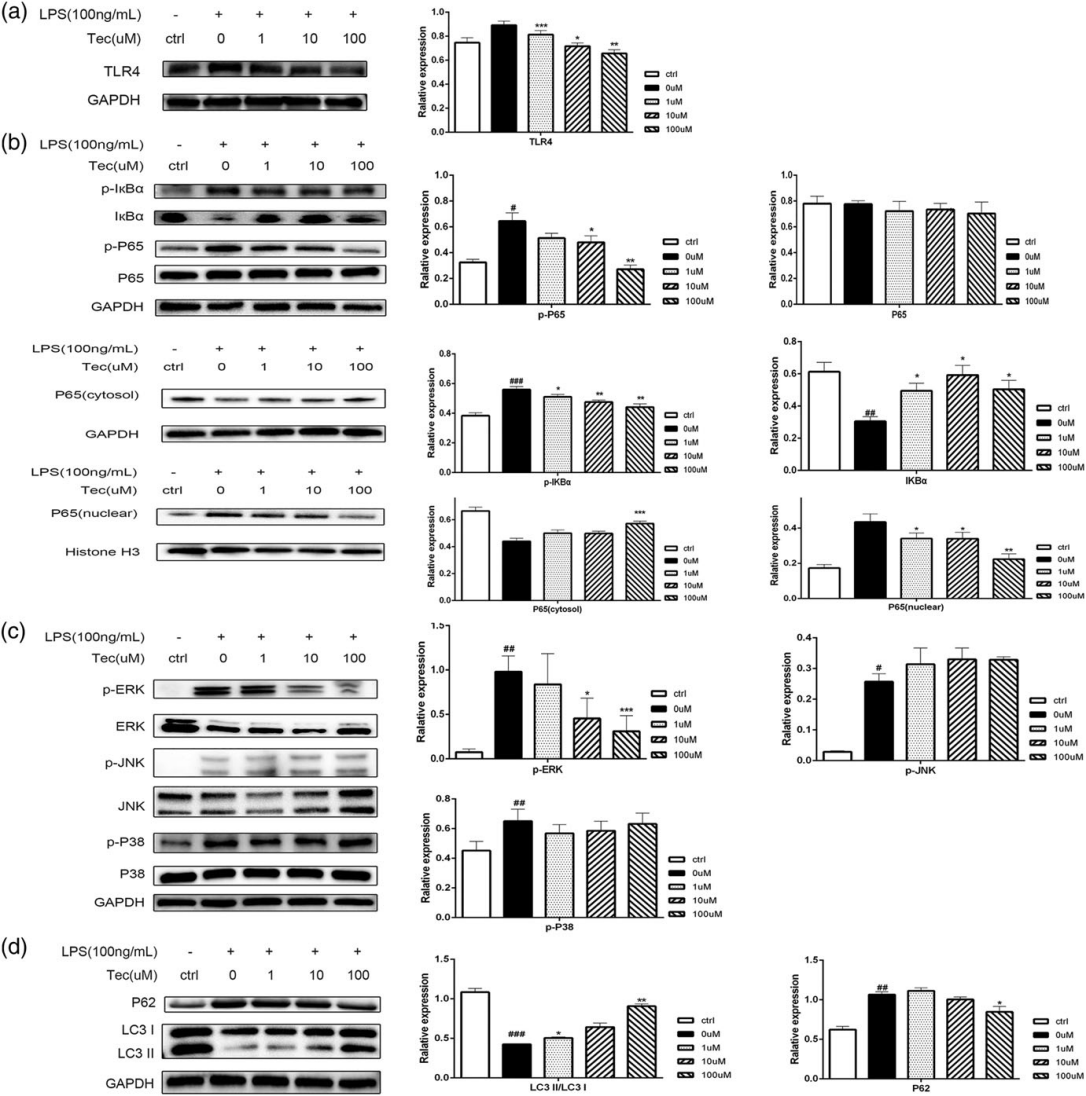
Tectorigenin blocks TLR4/mitogen‐activated protein kinase and TLR4/nuclear factor‐κB pathway activation and promotes autophagy in RAW 264.7 cells. Levels of (a) TLR4; (b) phosphorylated and total p65 and IκBα, and cytosolic and nuclear p65; (c) phosphorylated and total ERK, p38, and JNK; and (d) LC3 I, LC3 II, and p62 in RAW 264.7 cells were determined by Western blotting (*p < 0.05, **p < 0.01, and ***p < 0.001 compared with the lipopolysaccharide group. #*p* < 0.05, ##*p* < 0.01, and ###*p* < 0.001 compared with the control group; *n* = 3)

We also verified the effects of Tec on the TLR4/MAPK and TLR4/NF‐κB pathways and on autophagy in LPS‐stimulated RAW 264.7 cells. Tec decreased the TLR4 levels in vitro (Figure 5a). NF‐κB p65 and IκBα phosphorylation were significantly increased in LPS‐treated cells, and total IκBα was significantly downregulated (Figure 5b). However, Tec decreased the phosphorylation of NF‐κB p65 and IκBα, and total IκBα was significantly increased (Figure 5b). The p65 protein was largely present in the cytosolic fraction in the control group. After LPS administration, most of the p65 protein translocated from the cytosol to the nucleus (Figure 5b). However, Tec remarkably blocked this translocation (Figure 5b). Then, we assessed the effects of LPS on the MAPK family protein. ERK1/2, P38, and JNK phosphorylation were significantly increased in LPS‐stimulated RAW 264.7 cells (Figure 5c). The phosphorylation of ERK1/2, but not that of p38 and JNK, was attenuated by Tec in RAW 264.7 cells (Figure 5c).

Here, we also analyzed the effects of Tec on macrophage autophagy. Autophagy‐related proteins were detected in RAW264.7 cells in vitro. Autophagy was suppressed by LPS in vitro (Figure 5d). Tec promoted the lipidation of LC3I to LC3 II and p62 protein degradation in vitro (Figure 5d). In summary, Tec could suppress the TLR4/MAPK and TLR4/NF‐κB pathways and promote autophagy in LPS‐stimulated RAW 264.7 cells.

## 4 DISCUSSION

FHF is a severe clinical condition with high mortality, and, to date, liver transplantation remains the most effective treatment (Halliday & Westbrook, 2017). However, the application of liver transplantation is limited due to the available donations (Halliday & Westbrook, 2017). Thus, it is of great interest to find a new therapeutic option for FHF. Here, we found that Tec could effectively protect against LPS/D‐GalN‐induced FHF and reduce mortality. Due to limited alternative medical therapies of FHF, Tec could be one of the effective drugs for the treatment of FHF. Furthermore, previous toxicological studies have shown that Tec is not toxic at doses up to 300 mg/kg in mice during 28‐day treatment (Ha le, Que do, Huyen do, Long, & Dat, 2013), and our in vitro experiments also demonstrate that Tec is not hepatoxic. Thus, we thought Tec would be a safe and effective treatment option for FHF mice, although there is still no clinical data for Tec that have been published.

The application of herbs in liver diseases is an important alternative therapy in Eastern medicine (Schuppan, Jia, Brinkhaus, & Hahn, 1999). However, owing to the lack of standardization and randomized clinical trials, the promotion of herbs has been restricted over the past years. Thus, there is an urgent need for new evidence of the efficiency of herbs in the treatment of liver diseases. Recently, some pure compounds extracted from herbs such as quercetin and emodin have been demonstrated to have favorable protective effects in FHF animal experiments (Peng et al., 2017; Yin et al., 2014). In the previous reported studies, pretreatment with Tec showed anti‐inflammatory effects in an acute lung injury model and hepatoprotective effects in chemical and oxidative models (H. U. Lee et al., 2005; H. W. Lee et al., 2003; Ma et al., 2014). We investigated the therapy potential of Tec in mice with LPS/D‐GalN‐induced FHF. In the present study, we successfully established a mouse model of FHF by intraperitoneal injection of LPS/D‐GalN. The mortality, ALT and AST activities, and apoptosis were significantly increased by LPS/D‐GalN. Moreover, the hepatic architecture was disrupted, accompanied by necrosis and severe tissue hemorrhage. Tec pretreatment significantly decreased the elevated circulating levels of proinflammatory cytokines that were increased by the administration of LPS/D‐GalN. Additionally, Tec pretreatment decreased the circulating levels of ALT and AST, normalized the liver structure, and diminished apoptosis in liver tissues.

To explore the underlying mechanism of Tec, we examined the TLR4/MAPK and TLR4/NF‐κB pathways. TLR4 signaling plays a key role in the pathogenesis of liver injury (Ben Ari et al., 2012), and TLR4 is the essential receptor for LPS in immune cells (Medzhitov, 2001). An increased TLR4 level is correlated with elevated mortality and LPS/D‐GalN‐induced liver damage (Ben Ari et al., 2012). In this study, the TLR4 levels were significantly increased in vivo, whereas elevated TLR4 levels were decreased by pretreatment with Tec. TLR4 activation initiates a proinflammatory response dependent on the activation of MAPK and NF‐κB signaling pathways (Takeuchi & Akira, 2010). In this study, the activation of the MAPK pathway was significantly increased in mice with LPS/D‐GalN‐induced FHF. Tec pretreatment suppressed the activation of the MAPK pathway in vivo. The NF‐κB signaling pathway was also successfully activated in vivo. However, Tec reduced the activation of the NF‐κB pathway in mice with LPS/D‐GalN‐induced FHF.

Numerous studies reported that autophagy can protect the liver from FHF by suppressing the inflammatory response (Han et al., 2016; Lin et al., 2014). Autophagy also protects against liver injury by suppressing caspase activation and apoptosis (Amir et al., 2013; Ni, Bockus, Boggess, Jaeschke, & Ding, 2012). Genetic deletion of hepatic *Atg7* contributes to hepatomegaly and hepatic cell swelling (Komatsu et al., 2005). In our study, autophagy was suppressed by LPS/D‐GalN in vivo, as the lipidation of LC3 I to LC3 II decreased. Tec pretreatment induced the lipidation of LC3 I to LC3 II in mice. Tec did not significantly influence p62 degradation in vivo. In the present study, we demonstrate for the first time that Tec could promote autophagy in LPS/D‐GalN‐induced FHF.

Some multicenter studies have demonstrated that the presence of the systemic inflammatory response syndrome in ALF is associated with a poor prognosis (Antoniades et al., 2008). Innate immune cells released inflammatory cytokines following exposure to certain stimulating factors in liver injury, and cytokine storms could be extremely fatal (Takeuchi & Akira, 2010; Youn, Lee, Choi, & Park, 2016). Our in vitro studies confirmed the anti‐inflammatory effects of Tec in LPS‐induced RAW 264.7 cells for the reduction of proinflammatory cytokine levels both in mRNA and the culture supernatant. Tec reduced the TLR4 protein level in vitro, and Tec pretreatment also suppressed the activation of the MAPK pathway, which was significantly up regulated in LPS stimulated RAW 2647 cells. Moreover, the NF‐κB signaling pathway was also successfully activated in vitro, which was reduced by Tec as well. More importantly, autophagy, which was suppressed by LPS in vitro through decreasing lipidation of LC3 I to LC3 II and elevating p62 expression, was promoted by Tec pretreatment. In a way, Tec pretreatment eliminated the inflammatory reaction that was induced by LPS in FHF models and reduced its mortality.

**Figure 6:**
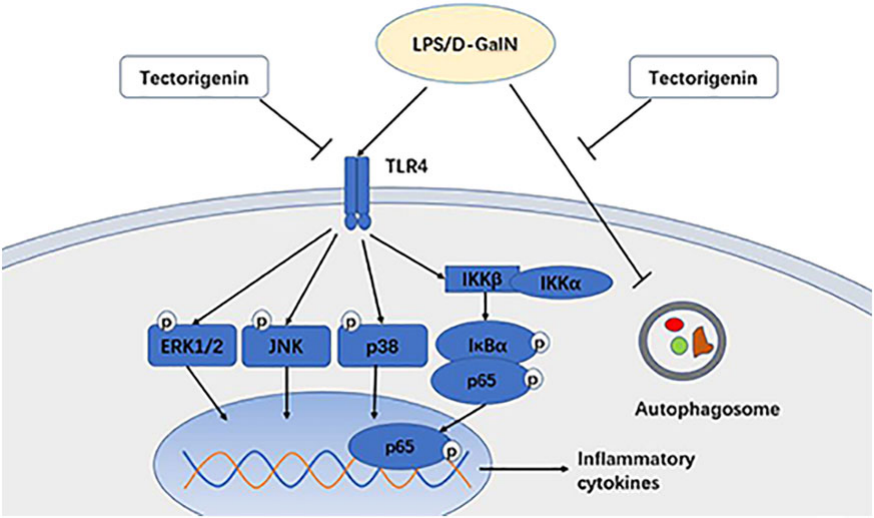
Potential schematic diagram of tectorigenin in LPS/D‐GalN‐induced fulminant hepatic failure. Our results suggest that tectorigenin has therapeutic potential for FHF in mice via the inhibition of TLR4/mitogen‐activated protein kinase and TLR4/nuclear factor‐κB pathways and promotion of autophagy. LPS/D‐GalN: lipopolysaccharide/D‐galactosamine‐GalN; JNK: c‐Jun N‐terminal kinase [Colour figure can be viewed at wileyonlinelibrary.com]

Although we have discovered that Tec could inhibit the TLR4/MAPK and TLR4/NF‐κB pathways and promote autophagy (Figure 6), specific molecular targets and related inflammatory pathways still need to be explored further. In addition, the possible function of Tec on the regulation of intestinal microecology in FHF is under study in our lab.

In conclusion, pretreatment with Tec attenuated hepatic inflammation, ameliorated liver injury, and reduced the mortality in mice with FHF by inhibiting inflammation via the TLR4/MAPK and TLR4/NF‐κB pathways and by promoting autophagy. Therefore, Tec is a promising drug for the treatment of FHF.

## CONFLICT OF INTEREST

The authors declare no conflict of interest.

## Supporting information

Figure S1. Effects of Tec on autophagosomes in LPS/D‐GalN‐induced FHF.
Table S1. The primers utilized for amplification of respective genes.
