# Supplementary material for: Tectorigenin protects against experimental fulminant hepatic failure by regulating the TLR4/mitogen‐activated protein kinase and TLR4/nuclear factor‐κB pathways and autophagy
Source: Phytother Res. 2019 Jan 30;33(4):1055–64. doi: 10.1002/ptr.6299 (PMC6590665; doi:10.1002/ptr.6299)
Supplement: Supplementary file 1 — Figure S1. Effects of Tec on autophagosomes in LPS/D‐GalN‐induced FHF. Table S1. The primers utilized for amplification of respective genes. [file PTR-33-1055-s001.docx]

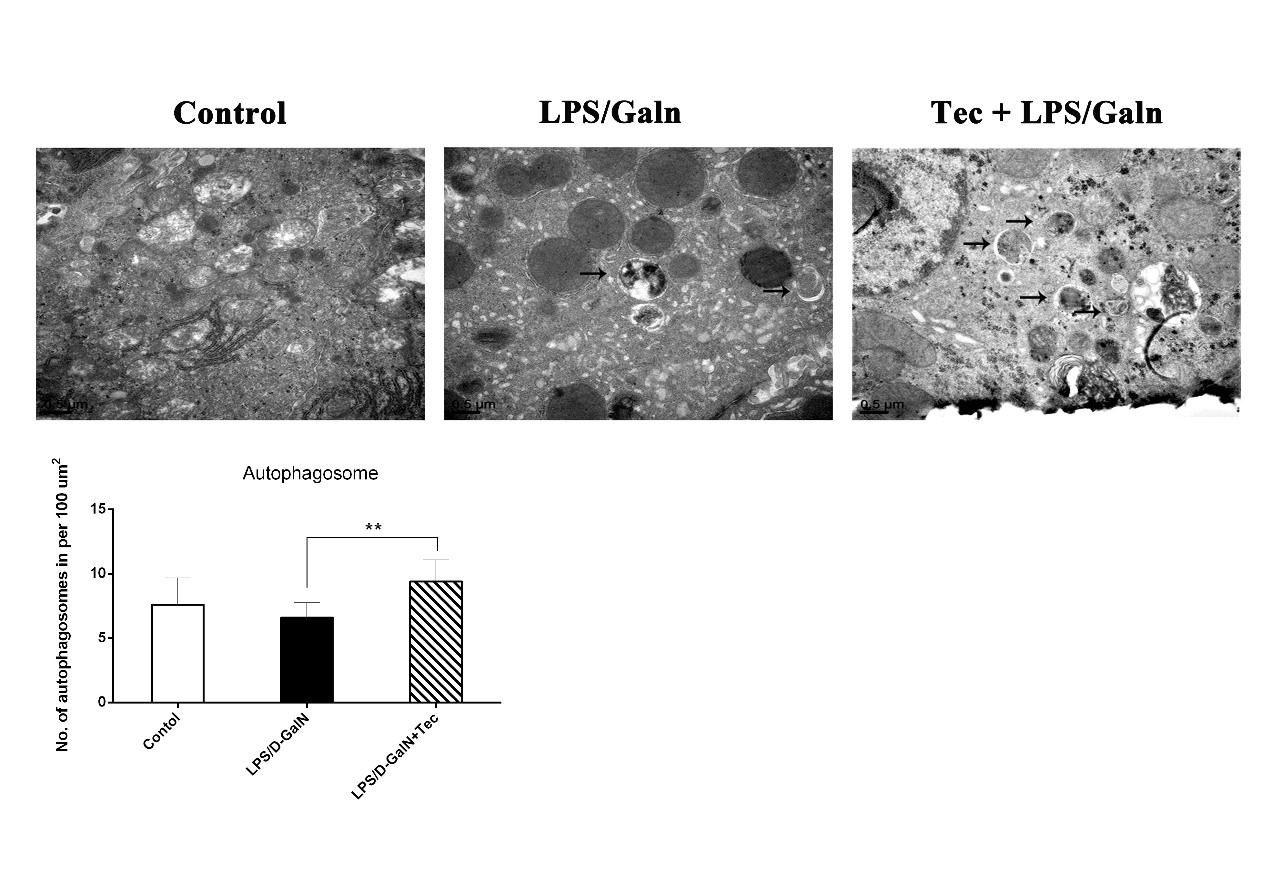
 **Figure S1. Effects of Tec on autophagosomes in LPS/D-GalN-induced FHF.** Liver samples at 6 h were processed for electron microscopy. Arrows denote autophagosomes. The number of autophagosomes was counted in per 100 um². (*p < 0.05 compared to the LPS/D-GalN group, n=5).

**Table S1. The primers utilized for amplification of respective genes.**

| Gene | Species | Forward primer (5’-3’) | Reverse primer (5’-3’) |
| --- | --- | --- | --- |
| actin | mice | GGCTGTATTCCCCTCCATCG | CCAGTTGGTAACAATGCCATGT |
| IL-1β | mice | TCGCTCAGGGTCACAAGAAA | CATCAGAGGCAAGGAGGAAAAC |
| IL-6 | mice | TCCATCCAGTTGCCTTCTTG | TTCCACGATTTCCCAGAGAAC |
| TNF-α | mice | AGGCTGCCCCGACTACGT | GACTTTCTCCTGGTATGAGATAGCAAA |
| COX2 | mice | TGAGCAACTATTCCAAACCAGC | GCACGTAGTCTTCGATCACTATC |
| IL-10 | mice | GCTCTTACTGACTGGCATGAG | CGCAGCTCTAGGAGCATGTG |
